# The CXCL12/CXCR7 signaling axis, isoforms, circadian rhythms, and tumor cellular composition dictate gradients in tissue

**DOI:** 10.1371/journal.pone.0187357

**Published:** 2017-11-08

**Authors:** Phillip C. Spinosa, Kathryn E. Luker, Gary D. Luker, Jennifer J. Linderman

**Affiliations:** 1 Department of Chemical Engineering, University of Michigan, Ann Arbor, Michigan, United States of America; 2 Department of Radiology, University of Michigan Medical School, Ann Arbor, Michigan, United States of America; 3 Department of Microbiology and Immunology, University of Michigan Medical School, Ann Arbor, Michigan, United States of America; 4 Department of Biomedical Engineering, University of Michigan, Ann Arbor, Michigan, United States of America; Katholieke Universiteit Leuven Rega Institute for Medical Research, BELGIUM

## Abstract

Chemokine CXCL12 gradients drive chemotaxis in a CXCR4-dependent mechanism and have been implicated in cancer metastasis. While CXCL12 gradients are typically studied in organized, defined environments, the tumor microenvironment is disorganized. *In vivo*, CXCL12 gradients depend on many factors: the number and arrangement of cells secreting and degrading CXCL12, isoform-dependent binding to the extracellular matrix, diffusion, and circadian fluctuations. We developed a computational model of the tumor microenvironment to simulate CXCL12 gradient dynamics in disorganized tissue. There are four major findings from the model. First, CXCL12-β and -γ form higher magnitude (steeper) gradients compared to CXCL12-α. Second, endothelial CXCR7+ cells regulate CXCL12 gradient direction by controlling concentrations near but not far from the vasculature. Third, the magnitude and direction of CXCL12 gradients are dependent on the local composition of secreting and scavenging cells within the tumor. We theorize that “micro-regions” of cellular heterogeneity within the tumor are responsible for forming strong gradients directed into the blood. Fourth, CXCL12 circadian fluctuations influence gradient magnitude but not direction. Our simulations provide predictions for future experiments in animal models. Understanding the generation of CXCL12 gradients is crucial to inhibiting cancer metastasis.

## Introduction

Chemokine CXCL12, alternatively called stromal cell-derived factor 1 (SDF-1), has been implicated in cancer metastasis and is a known driver of chemotaxis via chemokine receptor CXCR4 [1–4]. Common sites of breast cancer metastasis, including bone, liver, and brain, express high levels of CXCL12 [3,5]. Staining data demonstrate spatial heterogeneity of CXCL12 in tumors [2,6], suggesting that CXCL12 gradients exist *in vivo*. In *in vivo* models, CXCR4 antagonist AMD3100 (Plerixafor) decreases primary tumor size and/or metastatic burden, but metastatic disease is not cured [7–9]. One theory of the metastatic mechanism is that cancer cells use dynamic chemokine gradients in their local environments to travel from primary to secondary tumor sites. CXCL12 gradient dynamics remain unclear, and yet understanding these gradients *in vivo* is crucial to grasping the mechanism that drives cancer cells along the metastatic cascade.

CXCL12 has two known receptors, CXCR4 and CXCR7, which are involved in responding to and forming the CXCL12 gradient, respectively [10–12]. Many cell types in tumors secrete CXCL12, most notably cancer-associated fibroblasts. When CXCL12 binds to CXCR4, the receptor is internalized and a cascade of intracellular signaling events commences, culminating in a chemotactic response, cell survival, and proliferation. However, when CXCL12 binds to CXCR7, the chemokine is rapidly internalized and degraded, facilitating gradient formation [12], and a chemotactic response does not result [13]. CXCR7 is expressed throughout the tumor and is substantially upregulated on tumor-associated vasculature [14–17]. Endothelial CXCR7 is a key regulator of systemic CXCL12 blood plasma levels, which is unsurprising due to its location [18]. However, the role of endothelial CXCR7 with regards to CXCL12 gradients in tissue is unknown. In fact, it is difficult to fathom how cancer cells use chemokine gradients to intravasate into blood vessels if endothelial CXCR7 is present to scavenge CXCL12. Therefore, we asked how, why, and when CXCL12 gradients could be directed towards blood vessels. In this work, we study CXCL12 gradients in tumor geometries that are likely to exist *in vivo* while simultaneously examining the role of endothelial CXCR7.

Multiple mechanisms govern CXCL12 gradients *in vivo*. As CXCL12 is secreted in the tumor environment, it diffuses away, binds to extracellular matrix (ECM), and is degraded by both cellular and extracellular means. Alternative splicing generates six isoforms of CXCL12 [19], three of which are found in notable abundance in tumors and other sites in the body: CXCL12-α, -β, and -γ [20]. The isoforms bind nonspecifically to ECM with different affinities based on numbers of positively charged amino acids. CXCL12-γ binds to ECM with the highest affinity, followed by CXCL12-β and CXCL12-α, respectively [21]; their secretion rates from fibroblasts follow an opposite trend, with CXCL12-α being the highest [22]. Binding to ECM increases the local concentration of chemokine while also affording protection from both extracellular and cellular degradation [23]. Protection from degradation creates a slower effective diffusivity, thus creating isoform-specific gradients in tissue. Our group has found that CXCR4+ cancer cell migration in 2D is CXCL12 isoform-specific [22,24], but isoforms have not been examined and compared in 3D settings. In addition, CXCL12 levels in all tissues tend to vary according to the circadian rhythm up to 2-fold throughout a 24-h period [25,26], implying that tissue-level gradients are also time-dependent on this scale. The effect of circadian processes on tumor development, progression, and response to therapy is listed as a recent National Cancer Institute Provocative Question [27]. Previously, we calculated that cancer cells respond to CXCL12 gradients as small as 0.002 nM/μm [22]; circadian fluctuations might cause gradients to fall above and below this value throughout the course of a day. Since CXCR7 rapidly scavenges CXCL12, we questioned whether endothelial CXCR7 could modulate the effects of circadian fluctuations in the blood due to location between a tissue producing the chemokine and the vasculature. These dynamics collectively impact CXCL12 gradients in tissue.

We developed a computational model to simulate CXCL12 gradients in a tumor. We capture the geometry, cellular environment, and blood dynamics of a small tumor section and examine the magnitude, direction, and time variation of CXCL12 gradients. By studying CXCL12 gradient dynamics in a relevant, *in vivo*-like setting computationally, we can learn the major drivers of gradient formation, in particular the roles that CXCL12 isoforms, endothelial CXCR7, tumor composition, and circadian rhythms play. We seek to understand whether these mechanisms can form gradients directed into the blood despite the presence of endothelial CXCR7. Finally, we identify future experiments that can test our predictions regarding the influence of these drivers on cell migration and metastasis.

## Methods

### Model overview

Our computational model simulates CXCL12 transport in the tumor microenvironment. Cells exist in discrete locations within tumors. We use a 3D square lattice grid environment to place cells in distinct grid compartments, which allows us to alter the tumor cellular composition and distribution. We use two cell types: CXCL12-secreting cells and CXCR7+ cells. These cells represent fibroblasts and CXCR7+ tumor cells, respectively. CXCR7+ cells are dispersed throughout the tumor or lining the blood vessel, which spans the center of the grid. Each grid compartment has a side length of 10 μm, and can hold at most a single cell. The entire grid is a 200-μm cube. We simulate CXCL12 secretion, diffusion, degradation, binding, a blood vessel that can act as a source of CXCL12 for the tissue, and circadian fluctuations to depict the tumor microenvironment. Model parameters are given in Table A in S1 File and equations are given in Table B in S1 File.

### CXCL12 secretion, diffusion, and extracellular degradation

CXCL12-secreting cells secrete CXCL12 into the grid compartment the cell occupies and the chemokine diffuses away. Cells in any given simulation only secrete one CXCL12 isoform. Diffusion is solved using an Alternating Direction Explicit method [28]. We use no flux boundary conditions on the edges of the grid because we assume our model captures the dynamics of a small piece of tumor tissue and is surrounded by repeating similar units. Extracellular degradation of chemokine occurs according to first-order kinetics [29]. The timestep for secretion, diffusion, and extracellular degradation is 0.1 s.

### CXCL12 binding to receptors and to ECM

Receptor-mediated CXCL12 uptake by CXCR7+ cells, i.e. specific binding at the cell surface and intracellular trafficking of CXCL12 and CXCR7, is described by a set of previously published ordinary differential equations (Tables C-E in S1 File) [22,30]. CXCR4+ cells, which can also take up CXCL12, may additionally influence CXCL12 gradients. However, we assume that most CXCL12 degradation in tumors occurs via CXCR7 for three reasons: 1) the affinity of CXCL12 is approximately two orders of magnitude greater for CXCR7 than CXCR4 [31,32], 2) CXCR7 is often colocalized with CXCL12 [33], and 3) CXCR7 and CXCR4 are expressed in approximately the same levels [14].

On every diffusion timestep (0.1 s), CXCL12 binds to ECM in its grid compartment according to first-order kinetics and characterized by dissociation constant K_D_ (Table B in S1 File) [22]. Each grid compartment is associated with a free and ECM-bound CXCL12 concentration. We assume CXCR7+ cells can bind free and ECM-bound CXCL12 with the same binding parameters. When calculating CXCL12 gradients, we take the difference of the sum of the free and bound concentrations across a distance.

### CXCL12 circadian fluctuation and transport across the blood vessel wall

The presence of CXCL12 circadian fluctuations *in vivo* is attributed to a time-dependent secretion rate from cells in the body. However, because we model only a small tumor volume (8x10^-3^ mm^3^), we assume that any CXCL12 secreted from cells has no significant effect on CXCL12 blood concentration. In contrast, blood can deliver CXCL12 *to* the portion of the tumor that we simulate because we cannot neglect the influence of CXCL12-secreting cells at sites far from the simulated tumor. We represent the CXCL12 secreted by distant cells by assuming a bulk blood CXCL12 concentration. The CXCL12 secretion rate from cells is time-dependent while the blood fluctuation is simultaneously impressed on the system. The blood concentration varies temporally but not spatially within the vessel. The circadian variation takes the form *y* = *A* cos[*B*(*t* − (*C* − *D*)] + *E*, which allows us to characterize the amplitude, period, intercept, and time offset of the CXCL12 blood dynamics and cellular secretion rate according to:

$$X(t)=\left(\frac{X_{max}-X_{min}}{2}\right)\;cos(2\pi f(t-(t_{start}-t_{max}))+\left(\frac{X_{max}+X_{min}}{2}\right)$$ (1)

where X is either the secretion rate or CXCL12 blood concentration, X_max_ and X_min_ are maximum and minimum values of X, f is the frequency, and t_start_ and t_max_ are the time of day to start the simulation and the time at which maximum CXCL12 secretion and blood levels are observed [25], respectively.

CXCL12 transport from blood to tissue occurs according to a mass transfer boundary condition as shown by:

$$\frac{dn}{dt}=pA(C_{blood}-C_{endo})$$ (2)

where n is the moles of CXCL12 transported from blood to tissue, p is the vascular permeability to CXCL12, A is the surface area of the blood vessel seen by endothelial grid compartments, and C_blood_ and C_endo_ are the blood concentration and tissue concentration just outside the blood vessel wall, respectively.

### 3D tumor-like geometries

In order to simulate a tumor microenvironment, we place cells in geometries that are likely to occur *in vivo* [2,6,14–17]. A blood vessel is centered on the grid and can deliver CXCL12 to the surrounding tissue. CXCL12-secreting cells are placed randomly throughout the grid. CXCR7+ cells fall into one of two classes: tissue (non-endothelial) CXCR7+ cells, which are scattered randomly throughout the grid, or endothelial CXCR7+ cells, which line the blood vessel wall. No cells are seeded inside the blood vessel. We use a baseline of 200 cells of each type unless otherwise specified. We assume no cell motion since CXCL12-secreting cells and CXCR7+ cells do not exhibit chemotactic mobility [17,34]. Cells such as macrophages, lymphocytes and T cells that are present in tumors are assumed to occupy any remaining grid compartments; our focus here is on cell types that most notably directly impact CXCL12 gradients. Because of the randomness in initial cell placement, we average our model outputs over five runs.

### Model implementation

We study CXCL12 gradients using the model components described above. We use our model in two distinct setups.

**Fig 1:**
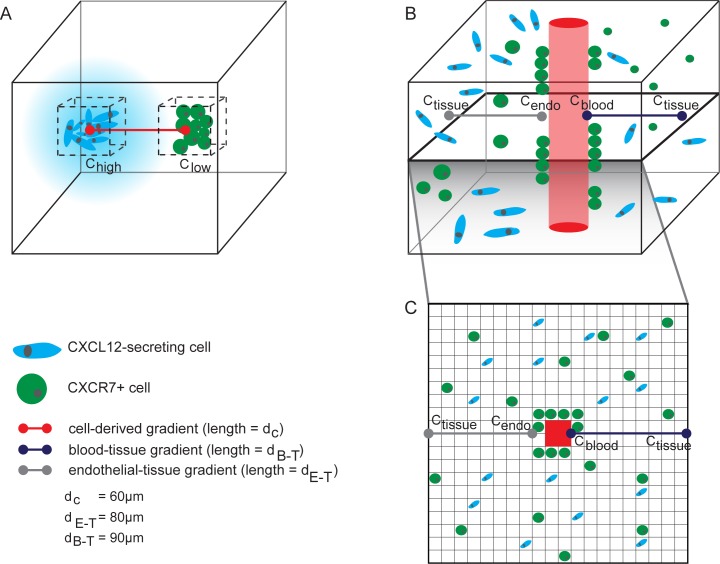
Model setups. **A**, In Setup 1, we capture the cell-derived CXCL12 gradient along the red line formed in the presence of clusters of CXCL12-secreting cells and CXCR7+ cells to examine the characteristics of isoform-specific gradients. **B**, In Setup 2, we simulate a section of a tumor and calculate the blood-tissue and endothelial-tissue gradients. All variables shown in figure are detailed in Appendix 2. **C,** A cross-sectional slice of the grid used for Setup 2.

In Setup 1 we examine CXCL12 isoform-specific gradients. CXCL12-secreting and CXCR7+ cells are placed in clusters 100 μm apart (Fig 1A). The clusters are confined to a cube region of side length equal to six cell diameters. We vary the number of cells and randomize their locations within each cluster while holding the confinement volume constant (bounded by black dotted lines in Fig 1A). The mechanisms in this setup are secretion, diffusion, extracellular degradation, and binding to ECM and CXCR7. There is no blood vessel and no circadian rhythm in these simulations; cells secrete at the baseline rate S (Table A in S1 File). When the simulation begins, CXCL12 gradients form and we track ligand concentrations throughout the grid. The outputs are the cell-derived CXCL12 gradient, the total amount of CXCL12 on the grid, and the time elapsed before steady state is reached. Steady state in this model setup is operationally defined as being reached when the cell-derived gradient changes by less than 0.1 nM/μm over 5 min.

In Setup 2 we examine *in vivo-*like CXCL12 gradients in the disorganized tumor microenvironment. We simulate a centralized blood vessel, cells in 3D tumor-like geometries, and circadian fluctuations (Fig 1B). The blood vessel is modeled as a rectangular prism with a cross-sectional area of 400 μm^2^. The blood vessel spans the entire height of the simulation space. A 2D slice of the grid is shown in Fig 1C. There are two stages to this simulation. First, we initialize the grid with CXCL12 to mimic a snapshot of the tumor environment at a single time point. To do this, we place cells and simulate CXCL12 secretion, diffusion, degradation, binding to ECM and CXCR7, and transport from blood to tissue until steady state. Circadian fluctuations in cell secretion rate and blood levels are not modeled during this initialization stage because they occur at the slowest time scale, on the order of hours. Second, we introduce circadian fluctuations, both in the secretion rate from cells and the CXCL12 blood levels. The output using this model setup is the time-dependent magnitude and direction of the CXCL12 gradient (See Appendix 2). We arbitrarily define positive gradients as situations where the CXCL12 concentration is higher in the tissue than in the blood, and negative gradients as situations where the CXCL12 concentration is higher in the blood than the tissue.

The model is coded in C++ and all subsequent analyses are performed using in-house MATLAB scripts (The MathWorks, Inc., Natick, MA). All simulations were performed on the University of Michigan’s HPC cluster.

### CXCL12 measurements in mice

The University of Michigan IACUC approved all animal procedures. To determine the effect of endothelial CXCR7 on CXCL12 circadian rhythms, we measured CXCL12 levels in plasma, femur, and tibia from C57BL/6 mice (WT) and mice with conditional deletion of CXCR7 from vascular endothelium (SCL Cre+) [35]. We collected 100 μL blood samples into tubes coated with 20mM EDTA. Samples were centrifuged at 16,100g for 10 minutes at 4°C to collect plasma. For the femur and tibia samples, we flushed each bone with 100 μL sterile PBS to harvest bone marrow. We centrifuged samples at 1,600g for 5 minutes to pellet bone marrow cells and collected the supernatant for ELISA. All samples were collected at the times indicated. The mice were housed with 12-hour light/dark cycle from 6am to 6pm. All samples were stored at -80°C prior to ELISA for CXCL12 (R&D Systems), performed by the University of Michigan Cancer Center Immunology Core. Mice were euthanized with a CO_2_ overdose.

## Results

### CXCL12-β and -γ are associated with longer gradient formation times, higher gradient magnitudes, and higher tissue concentrations than CXCL12-α

**Fig 2:**
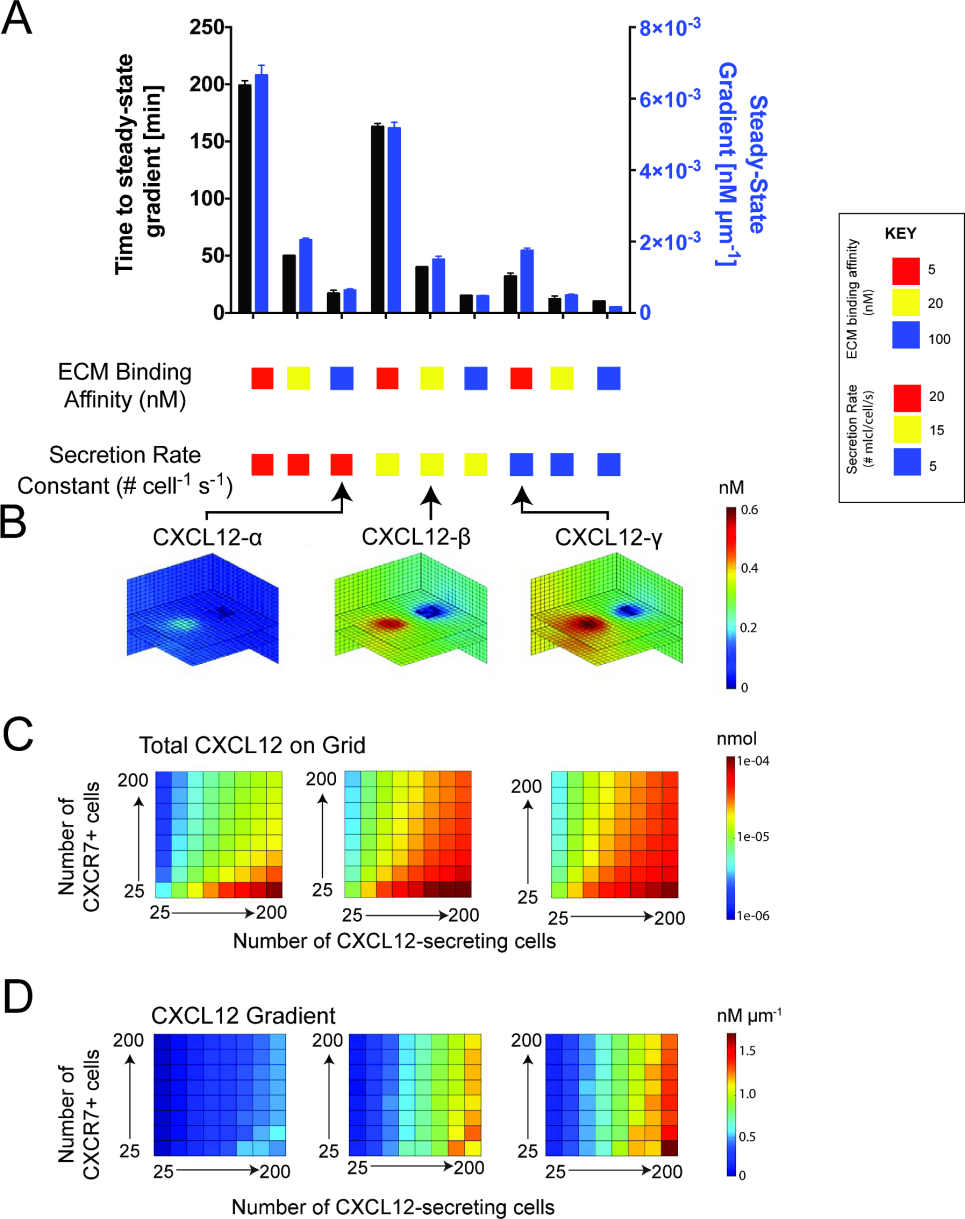
Cell-derived CXCL12 gradients are isoform-specific with regards to the time to steady state, gradient magnitude, and amount of CXCL12 in the proximate environment. **A**, Higher ECM binding affinity and higher secretion rate correspond to a higher gradient magnitude, but a longer time to form it. Error bars represent SEM of 5 simulations. Both clusters contain 100 cells. Cell clusters are placed 100 μm apart. **B**, CXCL12 gradients and total concentrations in the tissue vary with each isoform. Cell numbers were the same as in A. **C**, CXCL12-β (center) and -γ (right) create microenvironments with higher CXCL12 concentrations than CXCL12-α (left). On each plot, the x- and y-axes correspond to the number of cells in each cluster: 25, 50, 75, 100, 125, 150, 175, 200. **D**, the number of CXCL12-secreting cells in a cluster influences gradients, while the effect of the number of CXCR7+ cells is less pronounced. The x- and y-axes are the same as in C. For C and D, free and ECM-bound CXCL12 was summed. For all simulations in this figure, an average over 5 simulations is reported.

CXCL12 isoforms differ in their secretion rates from cells as well as their binding to ECM. We investigated how isoforms can differentially modulate gradients in a 3D environment. Using Setup 1, we placed a cluster of CXCL12-secreting cells and a cluster of CXCR7+ cells in our simulation (Fig 1A). We varied the CXCL12 secretion rate and CXCL12-ECM binding affinity to encompass known values for the α, β, and γ isoforms. As the secretion rate or the affinity increased, the cell-derived CXCL12 gradient magnitude increased (Fig 2A), consistent with published 2D simulations of a microfluidic source-sink device [22]. In addition, the time to reach steady state also increased. CXCL12-γ was able to maintain higher concentrations near source cells because of the lower effective diffusivity caused by binding to the ECM (Fig 2B). For each isoform, greater numbers of scavenging cells decreased the total amount of CXCL12 on the grid (Fig 2C) but had little effect on the magnitude of the gradient between the clusters (Fig 2D). Because the binding affinity of CXCL12 for CXCR7 is high, just a few CXCR7+ cells can degrade nearly all of the nearby CXCL12, so additional CXCR7+ cells have little effect. As the number of CXCL12-secreting cells increased, the amount of CXCL12 on the grid and gradient magnitudes increased, indicating that the number of source cells can have a substantial impact on gradients (Fig 2C and 2D). Taken together, these data imply that cell-derived CXCL12-β and -γ within tumor tissue form stronger gradients and create environments richer in CXCL12 than cell-derived CXCL12-α.

### Endothelial CXCR7 decreases CXCL12 levels during circadian fluctuations

**Fig 3:**
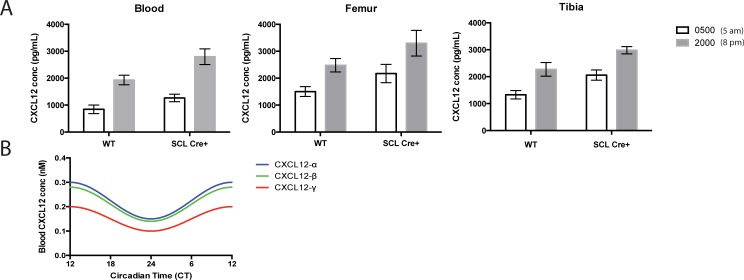
CXCL12 circadian fluctuations in CXCL12 concentration occur independent of endothelial CXCR7. **A**, Blood (left), femur (center), and tibia (right) CXCL12 levels vary 2-fold between 5 am and 8 pm. Error bars represent SEM of 5 repeated measurements per group of mice. **B**, CXCL12 isoform-specific human blood variations that we use in our model (Eq (1)). CT 24 corresponds to 9 pm while CT 12 corresponds to 9 am. For humans, we offset the time at which the maximum and minimum blood levels occur in the mice by 12 hours because mice are nocturnal. Mice and humans exhibit CXCL12 blood level maxima at night and in the morning, respectively.

Before moving on to tumor microenvironment simulations, we wanted to understand the interdependence of circadian fluctuations in CXCL12 concentrations and endothelial CXCR7 cells. CXCL12 secreted from cells must bypass endothelial CXCR7 cells to enter the blood, and endothelial CXCR7 regulates systemic CXCL12 plasma levels [18]. We questioned whether endothelial CXCR7 may disrupt circadian fluctuations. We measured CXCL12 levels at 5 am (0500) and 8 pm (2000) in WT and SCL Cre+ mice, from which we inducibly deleted CXCR7 from vascular endothelium. In the blood, femur, and tibia, CXCL12 levels were 2-fold higher at 8 pm compared to 5 am in both WT and SCL Cre+ mice, indicating circadian fluctuations are independent of endothelial CXCR7. As expected, SCL Cre+ mice showed higher levels of CXCL12 in the blood, femur, and tibia compared to WT (Fig 3A). Based on these data, we incorporated a 2-fold cosinusoidal variation in the CXCL12 blood level over 24-h according to Eq (1) in our model. Our computational model is calibrated to human data rather than mice. We consulted literature to determine human CXCL12 levels [36]. The amount of each CXCL12 isoform in the blood follows the same trend as the cellular secretion rate: CXCL12-α > CXCL12-β > CXCL12-γ. We use human time-dependent CXCL12 blood levels in our model (Fig 3B). Given these data, we are equipped to include circadian fluctuations and endothelial CXCR7+ cells in our model of the tumor microenvironment.

### Endothelial CXCR7 influences CXCL12 gradient direction by regulating concentrations near but not far from the vasculature

**Fig 4:**
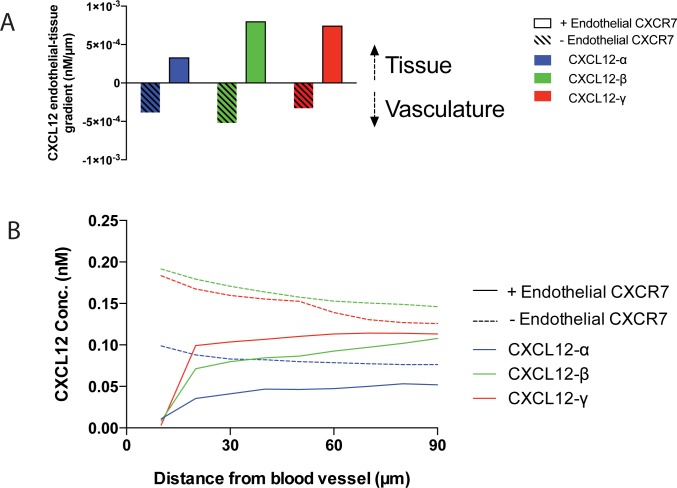
Endothelial CXCR7 influences CXCL12 gradient direction by controlling CXCL12 concentrations near but not far from the vasculature. 200 endothelial CXCR7 cells (83% coverage of the blood vessel), 200 CXCL12-secreting cells, and 200 CXCR7+ cells in tissue were placed in the simulation. **A**, Locally, endothelial CXCR7+ cells scavenge CXCL12 and influence the endothelial-tissue gradient direction. We arbitrarily define positive gradients as those that point from the vasculature to the tissue, and negative gradients point from the tissue to the vasculature. Maximum gradients over a 24-hour simulation are shown. **B**, CXCL12 concentration profiles (corresponding to the maximum gradients shown in A) indicate endothelial CXCR7 significantly decreases concentrations near the blood vessel and has a much lower effect far away. Curves begin at 10μm because this designates the first grid compartment next to the vasculature (Setup 2, Fig 1B and 1C).

Tumor-associated vasculature expresses endothelial CXCR7 that scavenges CXCL12. We investigated the ability of endothelial CXCR7 to control the direction of the CXCL12 gradient. We ran simulations of the tumor microenvironment (Setup 2, Fig 1B and 1C) for 24 hours and included two circadian sources of CXCL12: CXCL12 in the blood and CXCL12 secreted by cells within the tissue. We ran simulations both with and without endothelial CXCR7+ cells present and monitored the maximum endothelial-tissue gradient direction. When endothelial CXCR7+ cells were absent, the endothelial-tissue gradient was negative, indicating CXCL12 levels were higher near the vasculature than deeper in the tissue, and the gradient points towards the vasculature. In contrast, when endothelial CXCR7+ cells were present, the endothelial-tissue gradient was positive, indicating CXCL12 levels were higher deeper in the tissue than near the vasculature, and the gradient points into the tissue (Fig 4A). The magnitudes of the gradients were isoform-dependent. The isoforms with higher ECM binding affinity (CXCL12-β and -γ) maintained higher concentrations in the tissue, as anticipated from the results of Fig 2. The concentration profile from the vasculature through the tissue demonstrates that endothelial CXCR7+ cells regulate CXCL12 levels near the blood vessel, but they have a much smaller effect farther (90 μm) away (Fig 4B). From these data, we conclude that endothelial CXCR7 regulates gradient directions by scavenging CXCL12 near but not far from the vasculature.

### Local tumor cellular composition influences both CXCL12 gradient magnitude and direction, while circadian fluctuations only influence gradient magnitude

**Fig 5:**
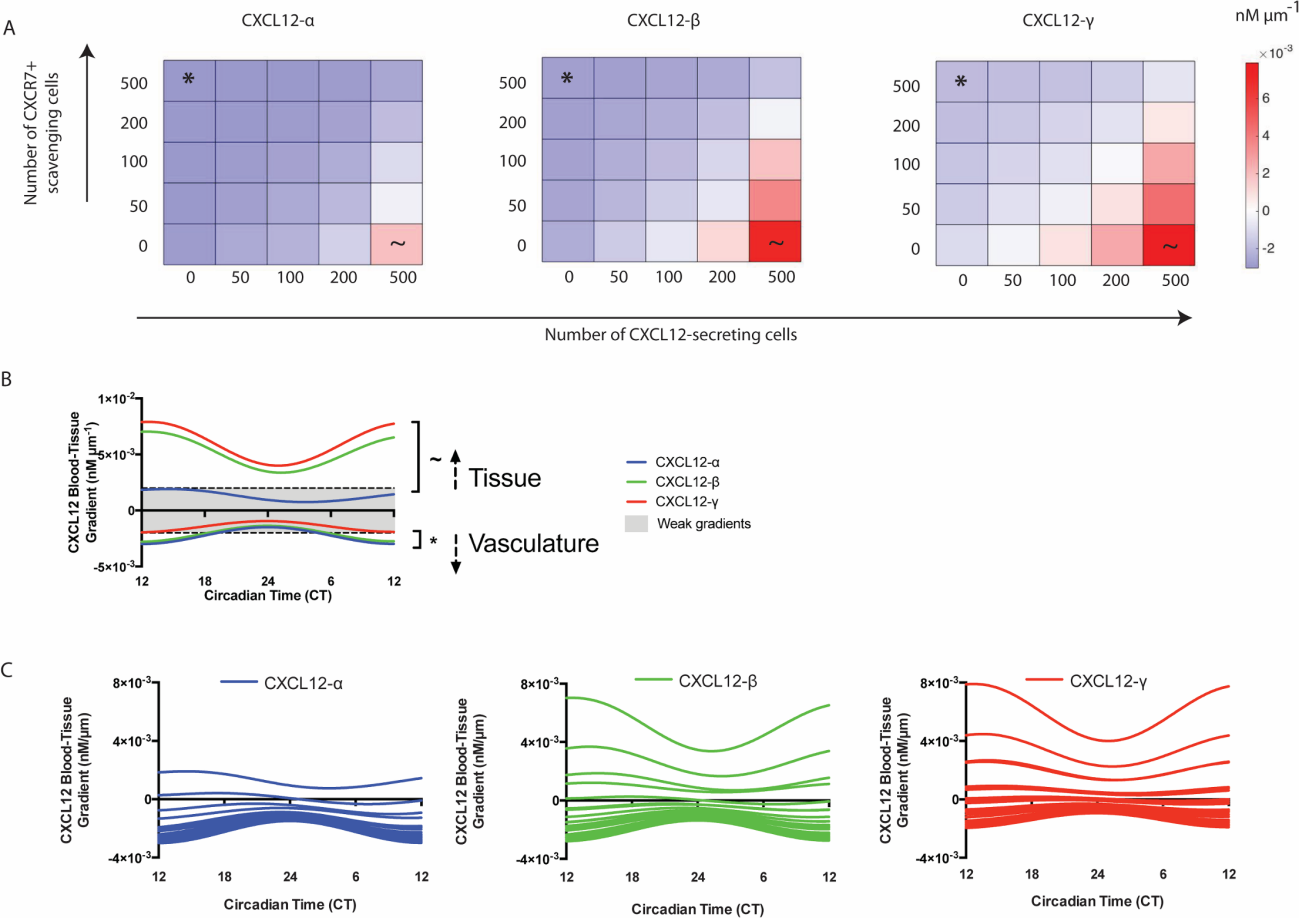
CXCL12-secreting and -scavenging cell numbers in the tumor microenvironment influence gradient direction and magnitude. **A**, Maximum CXCL12-α (left), -β (center), and -γ (right) gradients over a 24-h period illustrate that gradients are dependent upon cellular makeup. The * and ~ show the strongest gradients into the blood and into the tissue, respectively, and correspond to the same symbols in B. Gradient magnitudes were averaged over 5 runs to account for potential variation in the randomization of cell placement in the model. In all simulations, 200 endothelial CXCR7+ cells were present. White squares designate a gradient of exactly 0. **B**, Time-series of CXCL12 gradients demonstrate that gradients fluctuate according to circadian rhythm. Dotted lines represent the minimum gradient (0.002 nM/μm) that we determined to be necessary to trigger significant cell migration in a previous work [21]. CT 24 corresponds to 9 pm while CT 12 corresponds to 9 am. Positive gradients correspond to higher CXCL12 tissue concentrations while negative gradients correspond to higher CXCL12 concentrations in the blood. **C**, Circadian fluctuations of all combinations of CXCL12-secreting and -scavenging cells shown in A.

Because endothelial CXCR7 influences CXCL12 gradients in tumors, we also questioned how CXCR7+ cells deeper within tissue influence gradient magnitude and direction. Using Setup 2 (Fig 1B and 1C), we varied the number of CXCL12-secreting and -scavenging cells (non-endothelial CXCR7+ cells) in the tumor simulation. In all cases, endothelial CXCR7+ cells were also present. Predicted CXCL12 blood-tissue gradients over a 24 hour period, both magnitude and direction, are shown in Fig 5. The maximum blood-tissue gradient observed over this time interval is depicted in Fig 5A. CXCL12-α gradients were mostly directed into the blood, except at high CXCL12-secreting and low CXCR7+ cell numbers (Fig 5A, left). CXCL12-γ gradients are somewhat more likely to be directed into the tissue (Fig 5A, right). CXCL12-β blood-tissue gradients lie between CXCL12-α and -γ (Fig 5A, center). Isoforms with higher ECM binding affinity can more easily form gradients directed into the tissue. Overall, these results demonstrate that the CXCL12 gradient direction and magnitude depend on the numbers of CXCL12-secreting and non-endothelial scavenging cells present as well as the CXCL12 isoform.

To examine whether circadian fluctuations influence CXCL12 gradient magnitude and direction, we plot the 24-h time course (Fig 5B) of the simulations showing the largest gradients (denoted by * and ~ in Fig 5A). We compare these gradients to those calculated in earlier work as likely to generate cell migration [22]. At high CXCL12-secreting cell and low CXCR7+ cell numbers (denoted by ~), CXCL12-β and -γ force strong gradients away from the vasculature and into the tissue at all times of day. Substantial CXCL12-α gradients never form with the same tumor cellular composition. These results are in agreement with the data presented in Fig 2, which suggest that isoforms with higher ECM binding affinity form stronger cell-derived gradients. In contrast, when the CXCL12-secreting cell numbers are low and the CXCR7+ cell numbers are high (denoted by *), both CXCL12-α and -β gradients toward vasculature are predicted to be strong enough to elicit cell migration between about 3am and 3pm. CXCL12-γ gradients are minimal with this tumor cellular composition. Circadian fluctuations influenced gradient magnitudes but could not reverse the direction of the gradient, as shown by the lack of intersection of the curves with the x-axis. Additional simulations of combinations of secreting and scavenging cell numbers shown in Fig 5A similarly demonstrate that the circadian rhythm impacts gradient magnitude but not direction (Fig 5C). The amplitude of curves shown in Fig 5C decreases as the gradient magnitudes approaches 0, implying that weaker gradients vary less throughout the day than stronger ones (Figure A in S2 File). Although the circadian rhythm was unable to affect gradient direction, it is a critical regulator of the time-dependent gradient magnitude.

## Discussion

Chemokine gradients may facilitate the migration of cancer cells from a primary tumor, a critical step in metastasis. In previous computational and experimental studies, we found that a CXCL12 gradient on the order of 0.002 nM/μm, which corresponds to a difference of 10–20 molecules across the cell diameter, may be large enough to drive cancer cell migration [22]. Thus, a critical question is whether such gradients are likely to develop in tumors, and whether gradients point toward or away from the vasculature. Tumor spatial heterogeneity in the location and number of CXCR7+ cells as well as differences among CXCL12-α, -β, and -γ secretion rates and ECM binding may affect the gradients. Here, we use a computational model to understand the generation of CXCL12 gradients and calculate both the magnitude and direction of those gradients in a tumor microenvironment over time.

We wondered whether circadian oscillations in CXCL12 levels, driven by periodic alterations in cellular secretion rates throughout the body and affecting blood concentrations, would generate gradients in tumor tissue. We hypothesized that perhaps the oscillations could even reverse the gradient direction in the tumor tissue, so the gradient could dynamically switch between being directed into the bulk tissue and directed into the vasculature over the course of 24 hours. However, we found that for a given tumor cellular composition, the gradient is unable to switch directions because the two competing sources of CXCL12, the blood and the secretion rate from cells in the tumor, vary in unison. As the blood level increases, the secretion rate in the tumor also increases, and the gradient does not reverse because both the blood and tissue are gaining CXCL12 simultaneously. Nevertheless, circadian fluctuations affect gradient magnitudes and are hence still important *in vivo*. In fact, circadian fluctuations may influence the dosing schedule of cancer therapeutics [37,38], especially those that target the CXCL12 gradient or signaling axis.

It is difficult to fathom how CXCL12 gradients can cause cancer cell migration into blood vessels because of the presence of endothelial CXCR7 on most tumor vasculature. Indeed, our simulations suggest that endothelial CXCR7 encourages the formation of gradients pointed into the tissue (Fig 4A and 4B). Yet, our work also predicts that tumor regions with low numbers of CXCL12-secreting cells and high numbers of CXCR7+ cells in the bulk tumor tissue, even in the presence of endothelial CXCR7+ cells, can allow the formation of gradients pointing into the vasculature. In these cases, a dominant source of chemokine is CXCL12 originating from distant sites in the body that is delivered to the tumor via the blood circulation. The notion that cancer cells within the tumor respond to gradients formed from chemokine delivered from outside the tumor as well as chemokine generated within the tumor has not been well studied. This potential mechanism for the formation of gradients directed into the vasculature is supported by a statistical bioinformatics study of The Cancer Genome Atlas, which concluded that low CXCL12 levels in breast tumors correlated with more aggressive disease [20]. Other recent work has shown that sustained CXCL12 expression in the primary tumor inhibits metastasis, and that elevated CXCR7 levels and lower CXCL12 expression were related to poor survival [39]. Our data combined with these observations suggest that inhibiting CXCR7 may be crucial to preventing metastasis.

Our simulations show that endothelial and non-endothelial CXCR7+ cells both scavenge CXCL12 and thus regulate concentrations near and far from the blood vessel, respectively. In particular, endothelial CXCR7 is predicted to assist in maintaining CXCL12 gradients directed into the tissue (Fig 4). This is consistent with Stacer et al., who determined that endothelial CXCR7 is protective against metastasis [35]. At the same time, our simulations suggest that non-endothelial CXCR7 levels also play an important role in gradient generation; as described earlier, high levels can result in gradients directed toward the vasculature. Additionally, CXCR7 plays a role in neo-angiogenesis [17,33,40]. The multiple roles for CXCR7 should be considered when designing and administering inhibitors for this receptor.

**Fig 6:**
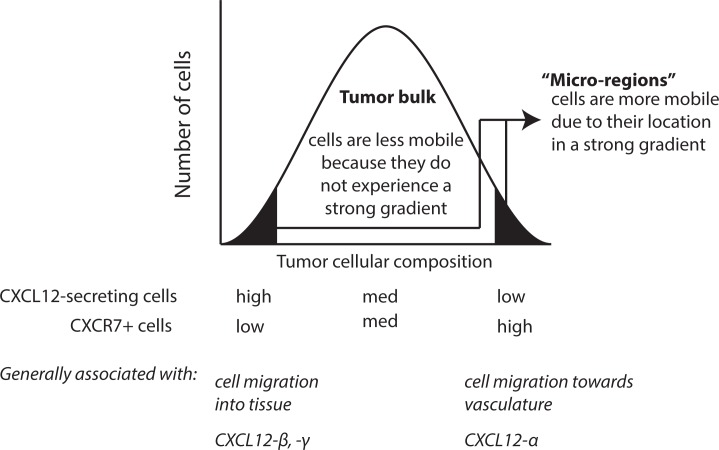
Cancer cells within “micro-regions” of the tumor containing either high or low ratios of CXCL12-secreting to non-endothelial CXCR7+ cells are more migratory than the tumor bulk. We assume tumors are mostly composed of immobile or weakly mobile cells due to a balance of CXCL12-secreting and CXCR7+ cells and a weak CXCL12 gradient. We predict that cancer cells located in environments containing a high number of CXCR7+ cells and few CXCL12-secreting cells will likely migrate towards vasculature, whereas cancer cells in environments with high secreting cell numbers and few CXCR7+ cells will likely migrate deeper into the tissue.

Tumors are notoriously heterogeneous [41–43]. There may exist “micro-regions” within a primary tumor that have a cellular composition that facilitates generation of strong gradients and therefore cell migration (Fig 6). Other areas of the same tumor without such conducive gradients would exhibit less cell migration [44]. As depicted in Fig 5, CXCL12 gradients in the tumor microenvironment are isoform dependent; isoforms with higher ECM binding affinity more easily form gradients directed into the tissue, whereas isoforms with lower ECM binding affinity more easily form gradients directed toward the vasculature. We thus predict that cells located in the permissive “micro-regions” account for the most migratory cells in the tumor and should be targeted to reduce metastatic burden.

Although we and others have shown that cell-derived CXCL12-γ forms gradients of higher magnitude *in vitro* than the -α isoform due to its lower effective diffusivity (Fig 2D) [21,22,24], here we predict that CXCL12-α can form stronger gradients directed toward the vasculature than the other isoforms *in vivo*. To explain this, consider a tumor region of high CXCR7+ cell and low CXCL12-secreting cell numbers. In this case, the blood is the dominant source of CXCL12, not cells in the tumor tissue. The blood concentration of CXCL12-α is noticeably higher than CXCL12-γ (Fig 3B). The high number of CXCR7+ cells in the tissue rapidly deplete CXCL12 regardless of isoform. Because the blood concentration of CXCL12-α is higher than CXCL12-γ and their tissue concentrations are nearly identical, the gradient directed into the vasculature is larger for CXCL12-α than -γ. This phenomenon is not seen in most *in vitro* chemotaxis studies because the specific *in vivo* setting, including the high ratio of scavenging to secreting cells, the large quantities of CXCL12 delivered from a separate source, and the relevant length scales, are necessary.

The predictions of our computational model suggest new *in vivo* experiments to study cancer cell migration and metastasis. We predict that mouse models containing tumors with CXCR4+ and CXCR7+ cells will cause more metastases than tumors containing only CXCR4+ cells (via analogy with Fig 6). As explained above, we also predict that CXCL12-α originating at distant sites far from the primary tumor will be better at forming gradients directed toward the vasculature than CXCL12-γ because of its higher blood concentration. By using our computational model to predict new experiments [45], we allow experimentalists to focus on studies with a high likelihood of revealing mechanisms of cancer cell migration and metastasis *in vivo*.

Our model predicts CXCL12 gradients *in vivo*, but has limitations. CXCL12 isoforms differ in their amino acid chain composition and length and therefore are likely differentially regulated by proteolytic degradation. When more quantitative information about the kinetics of these processes is available, the model can be used to assess any additional impact on CXCL12 gradients. In addition, CXCR4+ cells can influence the CXCL12 gradient, although minimally, and also signal differently through each isoform. Connell et. al found that CXCL12-γ binds sulfated tyrosine in N-terminus of CXCR4, which prevented CXCR4 activation [46]. Isoform-specific signaling through CXCR4 likely has implications for cell migration, and should be incorporated in models which include cell migration due to CXCL12 gradients.

As we learn more about the molecular-, cellular-, and tissue-scale mechanisms that drive the formation of CXCL12 gradients and presumably cancer metastasis, we will be more equipped to disrupt the process. The results of this study provide motivation that CXCL12 isoforms, the cellular composition of a tumor, and circadian rhythm fluctuations dynamically influence CXCL12 gradients. By providing an approach to define and analyze spatial and temporal dynamics of CXCL12 in tumors, we anticipate this research will help successfully advance CXCR7-targeted therapies into clinical oncology.

## Supporting information

S1 File Model details. (DOCX)

S2 File Correlation between the amplitude of the time-dependent blood-tissue gradient and the maximum observed gradient. (DOCX)
