# Supplementary material for: The CXCL12/CXCR7 signaling axis, isoforms, circadian rhythms, and tumor cellular composition dictate gradients in tissue
Source: PLoS One. 2017 Nov 8;12(11):e0187357. doi: 10.1371/journal.pone.0187357 (PMC5678865; doi:10.1371/journal.pone.0187357)
Supplement: S1 File — (DOCX) [file pone.0187357.s001.docx]

**The CXCL12/CXCR7 signaling axis, isoforms, circadian rhythms, and tumor cellular composition dictate gradients in tissue**

Phillip C Spinosa, Kathryn E Luker, Gary D Luker, Jennifer J Linderman

**S1 File. Supporting Information**

**S1 Table A.** Model Parameters

**S1 Table B.** Model Equations

**S1 Table C.** Molecular species involved in CXCR7-CXCL12 binding and trafficking events.

**S1 Table D.** Parameters describing CXCR7-CXCL12 binding and trafficking events.

**S1 Table E.** Ordinary differential equations which govern CXCR7-CXCL12 binding and trafficking events.

**S1 Table A.** Model Parameters

| **Category** | **Symbol** | **Description** | **Value** | **Units** | **Ref.** |
| --- | --- | --- | --- | --- | --- |
| *Blood* | p | CXCL12 permeability through blood vessel wall | 4.08x10^-5^ | cm s^-1^ | [1] |
|  | A | surface area of blood vessel bordering each endothelial compartment | 100 | μm^2^ |  |
|  | t_max_ | time of day of maximum CXCL12 blood concentration | 9am, converted to military time |  | [2], this work |
|  | t_start_ | time of day to start the simulation | arbitrary |  |  |
|  | C_CXCL12,max_ | maximum CXCL12 concentration in blood during circadian fluctuation | 0.30 (CXCL12-α)  0.28 (CXCL12-β)  0.20 (CXCL12-γ) | nM | [3] |
|  | C_CXCL12,min_ | minimum CXCL12 concentration in blood during circadian fluctuation | 0.15 (CXCL12-α)  0.14 (CXCL12-β)  0.10 (CXCL12-γ) | nM | [2] |
|  | f | frequency of circadian rhythm | 24 | h^-1^ |  |
| *Initialization thresholds* | BTIS_threshold_ | blood-tissue interface stability threshold | 1x10^-5^ |  |  |
|  | GS_threshold_ | gradient stability threshold | 1x10^-5^ |  |  |
| *Isoform-specific parameters* | K_D_ | binding affinity of CXCL12 for ECM | 100 (CXCL12-α)  20 (CXCL12-β)  5 (CXCL12-γ) | nM | [4] |
|  | S | baseline CXCL12 secretion rate from cells (Setup 1) | 20 (CXCL12-α)  15 (CXCL12-β)  5 (CXCL12-γ) | # (cell s)^-1^ | [4] |
|  | S_max_ | maximum CXCL12 secretion rate from cells during circadian fluctuation (Setup 2) | 20 (CXCL12-α)  15 (CXCL12-β)  5 (CXCL12-γ) | # (cell s)^-1^ | [4] |
|  | S_min_ | minimum CXCL12 secretion rate from cells during circadian fluctuation (Setup 2) | 10 (CXCL12-α)  7.5 (CXCL12-β)  2.5 (CXCL12-γ) | # (cell s)^-1^ | [2] |
| *Timesteps* | t_d_ | timestep for diffusion | 0.1 | s | [4] |
|  | t_m_ | timestep for receptor-ligand dynamics | 0.01 | s | [4] |
| *Other* | Φ | number of CXCL12 binding sites on ECM | 6.63x10^4^ | # compartment^-1^ | [4] |
|  | k_deg_ | Extracellular CXCL12 degradation rate | 2.05x10^-5^ | s^-1^ | [4] |
|  | D | CXCL12 diffusivity | 1.5x10^-6^ | cm^2^ s^-1^ | [5,6] |
|  | k_on_ | on-rate of CXCL12 to ECM | 0.001 | s^-1^ | [4,7] |

1. Zervantonakis IK, Hughes-Alford SK, Charest JL, Condeelis JS, Gertler FB, Kamm RD. Three-dimensional microfluidic model for tumor cell intravasation and endothelial barrier function. Proc Natl Acad Sci. 2012;109(34):13515–13520. doi:10.1073/pnas.1210182109.
2. Méndez-Ferrer S, Lucas D, Battista M, Frenette PS. Haematopoietic stem cell release is regulated by circadian oscillations. Nature. 2008;452(7186):442-447. doi:10.1038/nature06685.
3. Łukaszewicz-Zając M, Mroczko B, Kozłowski M, Szmitkowski M. The Serum Concentrations of Chemokine CXCL12 and Its Specific Receptor CXCR4 in Patients with Esophageal Cancer. Dis Markers. 2016;2016:7963895. doi:10.1155/2016/7963895.
4. Chang SL, Cavnar SP, Takayama S, Luker GD, Linderman JJ. Cell, Isoform, and Environment Factors Shape Gradients and Modulate Chemotaxis. PLoS One. 2015;10(4):e0123450. doi:10.1371/journal.pone.0123450
5. Lin F, Butcher EC. T cell chemotaxis in a simple microfluidic device. Lab Chip. 2006;6(11):1462–1469. doi:10.1039/b607071j.
6. Torisawa YS, Mosadegh B, Bersano-Begey T, Steele JM, Luker KE, Luker GD, et al. Microfluidic platform for chemotaxis in gradients formed by CXCL12 source-sink cells. Integr Biol (Camb). 2010;2(11-12):680–686. doi:10.1039/c0ib00041h.
7. Laguri C, Sadir R, Rueda P, Baleux F, Gans P, Arenzana-Seisdedos F, et al. The novel CXCL12gamma isoform encodes an unstructured cationic domain which regulates bioactivity and interaction with both glycosaminoglycans and CXCR4. PLoS One. 2007;2(10):e1110. doi:10.1371/journal.pone.0001110.

**S1 Table B**. Model Equations

| **Name** | **Equation** | **Symbols** |
| --- | --- | --- |
| Cell-derived CXCL12 gradient (G_C_) | $G_{C}=\frac{C_{high}-C_{low}}{d_{c}}$ | - C_high_ and C_low_ shown in Fig 1  - d_c_ = distance between clusters (100 μm) |
| Endothelial-tissue CXCL12 gradient (G_E-T_) | $G_{E-T}=\frac{C_{tissue}-C_{endo}}{d_{E-T}}$ | - C_tissue_ and C_endo_ shown in Fig 1  - d_E-T_ = horizontal distance from endothelium to edge of simulation space (80 μm) |
| Blood-tissue CXCL12 gradient (G_B-T_) | $G_{B-T}=\frac{C_{tissue}-C_{blood}}{d_{B-T}}$ | - C_tissue_ and C_blood_ shown in Fig 1  - d_B-T_ = horizontal distance from blood to edge of simulation space (90 μm) |
| Nonspecific CXCL12 binding to ECM | $\frac{dB}{dt}=k_{on}(\left[ CXCL12 \right]\left[ \phi\right]-K_{D}\left[ B \right])$ | - B = CXCL12 bound to ECM  - k_on_ = on-rate of CXCL12 to ECM  - K_D_ = binding affinity of CXCL12 for ECM  - Φ = empty sites on ECM |

**S1 Table C.** Molecular species involved in CXCR7-CXCL12 binding and trafficking events.

| **CXCR7 Species** | **Description** |
| --- | --- |
| *R_7_* (#/cell) | Free cell-surface CXCR7 |
| *L_12_* (nM) | Free extracellular CXCL12 |
| *B_e_* (#/cell) | Free endogenous β-arrestin 2 |
| *R_7Be_* (#/cell) | *R­_7_* bound to *B_e_* |
| *C_7_* (#/cell) | *R­_7_*  bound to *L_12_* |
| *C_7Be_* (#/cell) | *R­_7Be_* bound to *L_12_* |
| *R_7Bei_* (#/cell) | Intracellular *R­_7Be_* |
| *C_7Bei_* (#/cell) | Intracellular *C_7Be_* |
| *R_7Beii_* (#/cell) | *R_7Bei_* after *B_e_* dissociation |
| *C_7Beii_* (#/cell) | *C_7Bei_* after trafficking to late endosomes |
| *C_7Bpii_* (#/cell) | *C_7Bpi_* after trafficking to late endosomes |
| *L_12i_* (#/cell) | Intracellular *L_12_* |

**S1 Table D.** Parameters describing CXCR7-CXCL12 binding and trafficking events.

| **Parameter** | **Description** | **Value** |
| --- | --- | --- |
| *k_f,L12,7_* (nM^-1^s^-1^) | Forward rate constant of *L_12_* binding *R_7_ /R­_7Be_* | 1.4 x10^-3^ |
|  |  |  |
| *k_f,B,7_* ((#/cell)^-1^s^-1^) | Forward rate constant of *B_e_* binding *R­_7_ /C_7_* | 1.4 x10^-8^ |
|  |  |  |
| *K_D,R7,L12_* (nM) | Equilibrium dissociation constant of *L_12_* binding *R_7_* | 0.84 |
|  |  |  |
| *K_D,R7,B_* (#/cell) | Equilibrium dissociation constant of *B_e_* from *R_7_* | 2.3 x10^6^ |
|  |  |  |
| *K_D,C7,B_* (#/cell) | Equilibrium dissociation constant of *B_e_* from *C_7_* | 6.5 x10^5^ |
|  |  |  |
| *k_e,R7B_* (s^-1^) | *R_7Be_* internalization rate constant | 3.9 x10^-3^ |
|  |  |  |
| *k_e,C7B_* (s^-1^) | *C­_7Be_* internalization rate constant | 2.1 x10^-3^ |
|  |  |  |
| *k_off,B,7_* (s^-1^) | Dissociation rate constant of *B_e_* from *R_7Bei_* | 2.5 x10^-3^ |
|  |  |  |
| *k_e,C7Bi_* (s^-1^) | Rate constant of trafficking of *C­_7Bei_* to late endosomes | 5.5 x10^-4^ |
|  |  |  |
| *k_rec,R7Bii_* (s^-1^) | *R_7Beii_* recycling rate constant | 1.1 x10^-3^ |
|  |  |  |
| *k_rec,C7Bii_* (s^-1^) | *C­_7Beii_* recycling rate constant | 2.8 x10^-4^ |
|  |  |  |
| *k_deg,L12i_* (s^-1^) | *L_12i_* degradation rate constant | 1.0 x10^-4^ |

**S1 Table E.** Ordinary differential equations which govern CXCR7-CXCL12 binding and trafficking events.

| Ligand binding to free receptors | $v_{2}=k_{f,L_{12},7}(\left[ R_{7} \right]\left[ L_{12} \right]-K_{D,R_{7},L_{12}}\left[ C_{7} \right])$ |
| --- | --- |
| Ligand binding to receptor-β-arrestin complexes | $v_{4}=k_{f,L_{12},7}(\left[ R_{7B_{e}} \right]\left[ L_{12} \right]-K_{D,R_{7B},L_{12}}\left[ C_{7B_{e}} \right])$ |
| β-arrestin binding to free receptors | $v_{8}=k_{f,B,7}(\left[ R_{7} \right]\left[ B_{e} \right]-K_{D,R_{7},B}\left[ R_{7B_{e}} \right])$ |
| β-arrestin binding to ligand-bound receptors | $v_{12}=k_{f,B,7}(\left[ C_{7} \right]\left[ B_{e} \right]-K_{D,C_{7},B}\left[ C_{7B_{e}} \right])$ |
| Internalization of cell surface receptor-β-arrestin complexes | $v_{16}=k_{e,R_{7B}}\left[ R_{7B_{e}} \right]$ |
|  | $v_{20}=k_{e,C_{7B}}\left[ C_{7B_{e}} \right]$ |
| Dissociation of  β-arrestin from internalized receptor-β-arrestin complexes | $v_{25}=k_{off,B,4}\left[ C_{4B_{e}i} \right]$ |
| Trafficking of internalized receptor-β-arrestin complexes to late endosomes | $v_{27}=k_{e,C_{7Bi}}\left[ C_{7B_{e}i} \right]$ |
| Recycling of internalized receptors | $v_{30}=k_{rec,R_{7Bii}}\left[ R_{7B_{e}ii} \right]$ |
|  | $v_{33}=k_{rec,C_{7Bii}}\left[ C_{7B_{e}ii} \right]$ |
| Degradation of $L_{12i}$ | $v_{37}=k_{deg,L_{12i}}\left[ L_{12i} \right]$ |

| $\frac{d[R_{7}]}{dt}=-v_{2}-v_{8}-v_{10}+v_{30}+v_{32}+v_{33}+v_{34}$ |
| --- |
| $\frac{d[R_{7B_{e}}]}{dt}=+v_{8}-v_{4}-v_{16}$ |
| $\frac{d[C_{7}]}{dt}=+v_{2}-v_{12}-v_{14}$ |
| $\frac{d[C_{7B_{e}}]}{dt}=+v_{4}+v_{12}-v_{20}$ |
| $\frac{d[R_{7B_{e}i}]}{dt}=+v_{16}-v_{23}$ |
| $\frac{d[C_{7B_{e}i}]}{dt}=+v_{20}-v_{27}$ |
| $\frac{d[C_{7B_{e}ii}]}{dt}=+v_{27}-v_{33}$ |
| $\sim\frac{d[L_{12}]}{dt}=(-v_{2}-v_{4}-v_{6})\times\frac{{10}^{9}\frac{nmol}{mol}}{V_{cell}\times N_{Av}}$ |
| $\frac{d[L_{12i}]}{dt}=+v_{20}+v_{22}-v_{37}$ |
| $\frac{d[B_{e}]}{dt}=-v_{8}-v_{12}+v_{23}+v_{33}$ |
| $\frac{d[R_{7B_{e}ii}]}{dt}=+v_{23}-v_{30}$ |

~ Units are nM/s

V_cell_ = volume of a cell, 1x10^-12^ L

N_AV_ = Avogadro’s Number, 6.022x10^23^ mlcl / mol

Nomenclature, parameters, and equations shown in S1 Tables 3, 4, and 5 are taken from:

Chang SL, Cavnar SP, Takayama S, Luker GD, Linderman JJ. Cell, Isoform, and Environment Factors Shape Gradients and Modulate Chemotaxis. PLoS One. 2015;10(4):e0123450. doi:10.1371/journal.pone.0123450
