# Supplementary material for: The CXCL12/CXCR7 signaling axis, isoforms, circadian rhythms, and tumor cellular composition dictate gradients in tissue
Source: PLoS One. 2017 Nov 8;12(11):e0187357. doi: 10.1371/journal.pone.0187357 (PMC5678865; doi:10.1371/journal.pone.0187357)
Supplement: S2 File — (DOCX) [file pone.0187357.s002.docx]

**The CXCL12/CXCR7 signaling axis, isoforms, circadian rhythms, and tumor cellular composition dictate gradients in tissue**

Phillip C Spinosa, Kathryn E Luker, Gary D Luker, Jennifer J Linderman

**S2 File. Supporting Information**

**
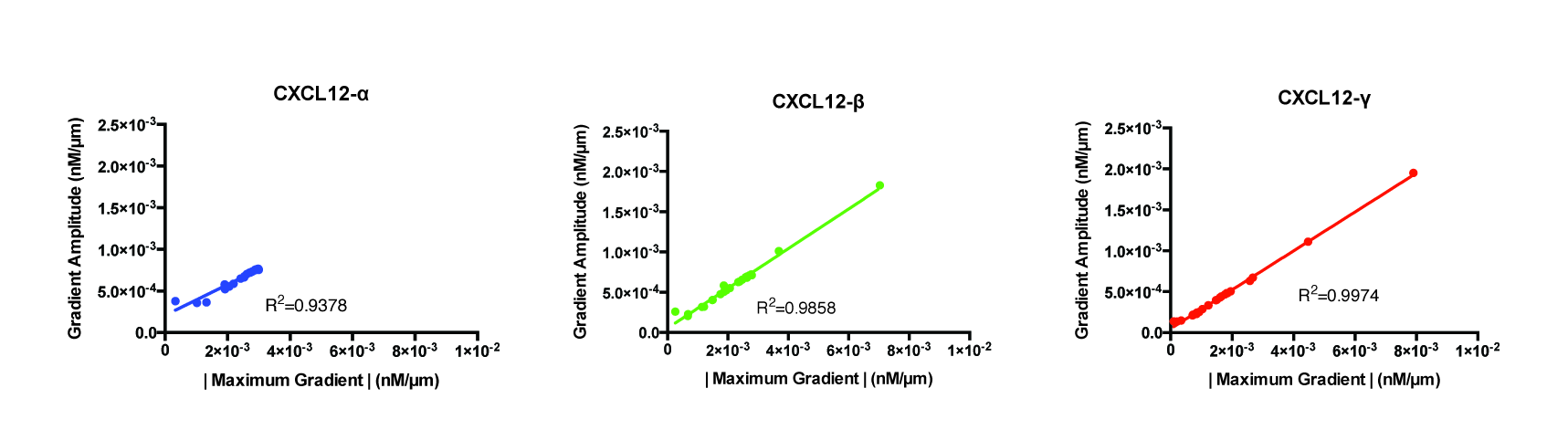
**

**S2 Fig A.** Correlation between the amplitude of the time-dependent blood-tissue gradient and the maximum observed gradient.

We ran our computational model of the tumor microenvironment and varied the number of CXCL12-secreting and CXCR7+ cells in tissue using the same values as in Fig 5A. We found that the amplitude of the blood-tissue gradient curve over 24 hours correlated with the maximum gradient in a linear trend for all isoforms.
